# Vitamin B6 Supplementation Improves Oxidative Stress and Enhances Serum Paraoxonase/Arylesterase Activities in Streptozotocin-Induced Diabetic Rats

**DOI:** 10.1155/2014/351598

**Published:** 2014-11-06

**Authors:** Sibel Taş, Emre Sarandöl, Melahat Dirican

**Affiliations:** ^1^Department of Biology, Science and Literature Faculty, Uludağ University, 16059 Bursa, Turkey; ^2^Department of Biochemistry, Medical Faculty, Uludağ University, 16059 Bursa, Turkey

## Abstract

The purpose of this study was to investigate the effects of vitamin B6 (Vit B6) on oxidant and antioxidant status in streptozotocin-induced diabetic rats. Thirty-two Wistar rats were divided into four groups: control (C), control + Vit B6 group (C + Vit B6), diabetes (D), and diabetes + Vit B6 group (D + Vit B6). Vit B6 (4 mg/kg body weight) was administered in drinking water for 4 weeks after the induction of diabetes. Vitamin B6 reduced serum total cholesterol level in the C + Vit B6 (*P* < 0.01) and D + Vit B6 (*P* < 0.05) groups. Plasma and tissue malondialdehyde levels were reduced in the C + Vit B6 and D + Vit B6 groups. Whole blood glutathione peroxidase (GSH-Px) and erythrocyte superoxide dismutase (SOD) activities were higher in the D group (*P* < 0.05). GSH-Px and SOD activities were increased in C + Vit B6 group while these parameters decreased in the D + Vit B6 group. Paraoxonase and arylesterase activities were decreased in the D group while they were increased in C + Vit B6 and D + Vit B6 groups. The results of present study suggest that vitamin B6 supplementation might be a promising adjunctive agent for improving oxidative stress and metabolic disturbances and for preventing diabetic complications including atherogenesis.

## 1. Introduction

Diabetes mellitus is a chronic metabolic disorder characterized by chronic hyperglycemia and disturbances of carbohydrate, lipid, and protein metabolism [1] those which result in several serious complications, including atherosclerotic vascular disease. Excessive production of reactive oxygen species (ROS) and/or their inadequate neutralization by antioxidants, defined as oxidative stress, leads to damage in cell membrane, vessel wall, proteins, lipids, and even the nucleic acids in the cell. Oxidative stress contributes to the progress of diabetic complications particularly atherogenesis [2, 3]. In normal conditions, to control the flux of ROS, aerobic cells have developed their own defense system, the antioxidant system that includes nonenzymatic (such as glutathione, A, C, and E vitamins) and enzymatic components (such as superoxide dismutase, glutathione peroxidase, and catalase) [4]. One of those antioxidant enzymes is paraoxonase (PON1) which is associated with high-density lipoprotein (HDL); PON1 was shown to protect lipoproteins, HDL, and low-density lipoprotein (LDL), from oxidation [5] which has been accepted to play a pivotal role in the development of atherosclerosis [6–8]. PON1 exerts both paraoxonase and arylesterase activities (ARE), since the enzyme hydrolyzes organophosphates (such as paraoxon) and hydrolyze aromatic esters (such as phenyl acetate) [9, 10]. It was reported that, related to ROS attack, PON1 and ARE enzyme levels and/or activity were decreased in states of high oxidative stress like diabetes, coronary artery disease, and dyslipidemia [11–13]. Some authors suggested that administration of antioxidants could protect PON1 from inactivation and/or diminishment arising from ROS and consequently ameliorate diabetes and related complications [14–16].

Vitamin B6, also called pyridoxine, is one of water-soluble B vitamins that assist in the metabolism of proteins, fats, and carbohydrates [17]. Pyridoxine, although not classified as a classical antioxidant compound, has recently been shown to have highly efficient antioxidant effects [18–21]. It was demonstrated that pyridoxine acts as a highly efficient hydroxyl radical (•OH) quencher with a capacity of scavenging up to eight •OH molecules [18–20]. Pyridoxine deficiency was also suggested to be associated with atherogenesis since it influences long-chain polyunsaturated fatty acids biosynthesis, increases lipid peroxidation, and affects antioxidant defense [21–23].

The number of studies about the effect of vitamin B6 on metabolic changes and oxidative stress in diabetes mellitus is limited [24–27]. In the present study, our aim was to investigate the effects of vitamin B6 on the lipid profile and oxidative and antioxidative system in streptozotocin- (STZ-) induced diabetic rats. To the best of our knowledge, there is no investigation about the effects of serum vitamin B6 on serum paraoxonase/arylesterase activities in diabetic rats. For this purpose, in order to evaluate the antioxidative defense system we measured serum paraoxonase and arylesterase activities, erythrocyte superoxide dismutase (SOD), and whole blood glutathione peroxidase (GSH-Px) activities. We determined plasma and tissue (musculus gastrocnemius, heart, liver, and kidney) malondialdehyde (MDA) levels so as to investigate the oxidative status of the rats. We also investigated blood lipid profile, including total cholesterol (TC), HDL-C, and triglyceride (TG) levels.

## 2. Materials and Methods

### 2.1. Animals

The experiments were performed with 32 male Wistar strain rats (age: 5 months) weighing approximately 300–350 g. The rats were housed at room temperature (25 ± 2°C) under a 12 h light-dark cycle. Rats were given free access to standard laboratory chow (carbohydrates 35%, proteins 25%, lipids 7%, and vitamins 3%) and tap water for one week before the experiment. Four rats were housed per cage. Experimental protocol was approved by the accordance with ethical procedures and policies approved by the Animal Care and Use Committee of Uludağ University, Bursa.

### 2.2. Experimental Design

The rats were divided into four groups of 8 rats each:

 Group I: normal control rats (C),
 Group II: control rats with orally administered vitamin B6 (C + Vit B6),
 Group III: STZ induced diabetic rats (D),
 Group IV: diabetic rats with orally administered vitamin B6 (D + Vit B6).

### 2.3. Diabetes Induction

Diabetes was induced with a single intraperitoneal injection of 65 mg/kg STZ (Sigma, St. Louis, MO) freshly dissolved in sodium citrate buffer (pH 4.5). Control rats received an injection of citrate buffer. Blood glucose level was measured 48 h after STZ injection. Rats with blood glucose levels >11 mmol/L were considered diabetic and were used for the study. Blood glucose concentration was measured with a Glucostix strip test in a glucometer (Abbott Glucometer Medisense Products, USA). Streptozotocin injection may result in fatal hypoglycemia related to massive insulin release. To prevent hypoglycemia, rats were kept on a 5% glucose solution diet for 24 h after the injection.

### 2.4. Vitamin B6 Treatment (Pyridoxine)

Vitamin B6 (Pyridoxine, Sigma) was prepared daily and administrated in drinking water at concentration (4 mg/kg body weight) to C + Vit B6 and D + Vit B6 groups for four weeks after STZ-injection.

### 2.5. Sample Preparation

At the end of the experimental period, blood samples were obtained by cardiac puncture under light ether anesthesia following 10–12 h of fasting. Liver, kidney, heart, and skeletal muscle (musculus gastrocnemius) tissues were removed immediately after blood collection, rinsed with cold saline, blotted with gauze, and stored at −20°C until analysis. Blood samples were drawn in heparin-coated, EDTA-containing, and nonadditive tubes. A part of whole blood was frozen for GSH-Px determination. Erythrocytes for SOD determination were washed by saline and frozen after hemolysis. Blood samples were stored at −20°C until analysis process.

### 2.6. Analyses

Paraoxonase activity was determined as described by Eckerson et al. [28]. The rate of hydrolysis of paraoxon was measured by monitoring the increase in absorbance at 412 nm at 25°C. Paraoxonase activity is expressed in U/L serum and defined as 1 mmol p-nitrophenol generated per minute under the above conditions. Arylesterase activity was determined by using phenylacetate as the substrate. The reaction mixture contained 1.0 mM phenylacetate and 0.9 mM calcium chloride in 9.0 mM Tris-HCl buffer, pH 8.0. One unit of arylesterase activity is defined as 1 mmol phenol generated per minute under the above conditions and expressed as kU/L serum [29]. Erythrocyte SOD and whole blood GSH-Px activities were determined using commercial kits (Randox Laboratories Antrim, UK). Briefly, the determination of SOD activity was based on the production of superoxide anions by the xanthine/xanthine oxidase system. GSH-Px catalyzed the oxidation of reduced glutathione in the presence of cumene hydroperoxide. The generation of nicotinamide adenine dinucleotide phosphate was measured spectrophotometrically at 340 nm [30]. Tissue MDA levels were determined by the thiobarbituric acid method and expressed as nmol MDA/mg tissue [31]. Plasma MDA concentrations were determined with the high-performance liquid chromatography (HPLC Shimadzu LC-10AT) procedure of Young and Trimble [32]. A calibration curve was prepared for each day by using 1,1′,3,3′tetraethoxypropane as the standard.

Sera TC, TG, and HDL-C levels were determined by standard laboratory methods using an autoanalyzer (Aeroset system Abbott, Abbott Laboratories Diagnostics Division, Chicago, IL, USA). Plasma vitamin B6 (pyridoxal and pyridoxal phosphate) concentration was measured by high-performance liquid chromatography (Thermo Finnigan Spectra Systems HPLC, USA).

### 2.7. Statistical Analysis

Data are presented as mean ± SEM. Kruskal Wallis test was used, followed by the Mann-Whitney *U* test. A level of *P* < 0.05 was accepted as statistically significant. Statistical analyses were carried out by SPSS 13.0 program for Windows.

## 3. Results

Table 1 shows features of diabetic and normal rats used in the present study. In the D group, food and fluid consumption, blood glucose, TC (*P* < 0.05), and TG levels (*P* < 0.01) were significantly increased, and final body weight (*P* < 0.05) significantly decreased compared with the C group. Serum TC levels were significantly decreased in C + Vit B6 (*P* < 0.01) and D + Vit B6 (*P* < 0.05) groups compared with the C and D groups, respectively. HDL-C levels were significantly increased in the C + Vit B6 (*P* < 0.01) and D + Vit B6 (*P* < 0.05) groups compared with those of the C and D groups, respectively. There was no difference in the final body weight, food, fluid consumption, and blood glucose levels of C + Vit B6 and D + Vit B6 groups compared with those of the C and D groups, respectively.

Concentrations of serum vitamin B6 values are shown in Table 2. Compared with the C group, serum vitamin B6 level was significantly decreased in the D group (*P* < 0.05); however, supplementation of vitamin B6 for 4 weeks increased serum vitamin B6 levels in C + Vit B6 and D + Vit B6 compared with the C and D groups, respectively (*P* < 0.05).

Serum paraoxonase and arylesterase activities in the D group were significantly decreased compared with those of the C group (*P* < 0.01); however, these parameters were significantly increased in the C + Vit B6 and D + Vit B6 group compared with the C and D groups, respectively (*P* < 0.05) (Table 2). Compared with the C group, whole blood GSH-Px and erythrocyte SOD activities were significantly higher in the D group (*P* < 0.05). Whole blood GSH-Px (*P* < 0.05) and erythrocyte SOD (*P* < 0.01) activities were increased in the C + Vit B6 but decreased in D + Vit B6 compared with the C and D groups, respectively (*P* < 0.05) (Table 2).

**Figure 1:**
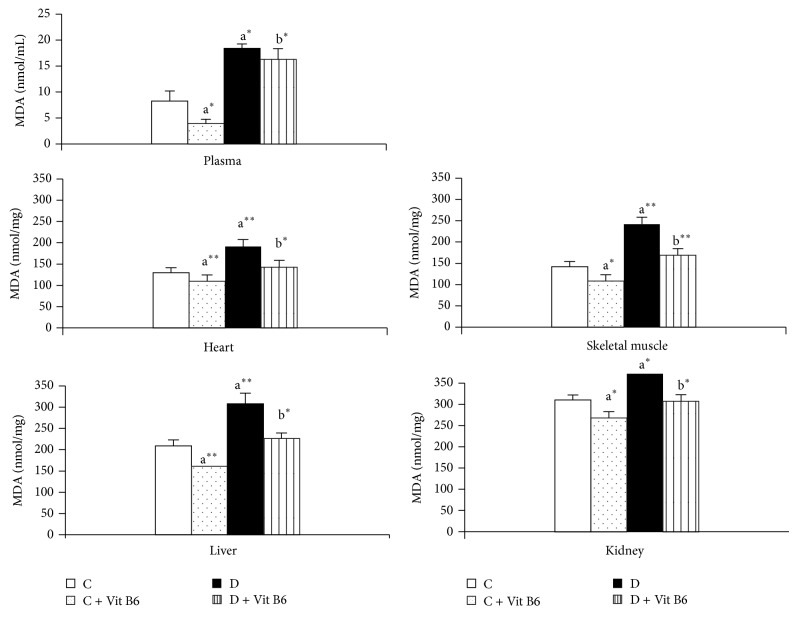
Malondialdehyde (MDA) levels in plasma (nmol/mL) and tissues (nmol/mg tissue) of the control and experimental rats. Values are expressed as mean ± SEM (standard error of mean) for eight rats in each group. Statistical comparison: ^a^compared with the C group, ^b^compared with the D group. Statistical significance, ^*^ *P* < 0.05 and ^**^ *P* < 0.01. For abbreviations of study groups; see Table 1.

As shown in Figure 1, plasma and kidney (*P* < 0.05), heart, skeletal muscle, and liver tissue MDA levels were significantly higher in the D group compared with the C group (*P* < 0.01). However, plasma and tissues MDA levels were decreased in the C + Vit B6 and D + Vit B6 groups compared with the C and D groups, respectively.

## 4. Discussion

In the present study, we demonstrated that STZ administrated rats were obviously diabetic and under oxidative stress as reflected by changes in body weight, food and fluid intake, and laboratory parameters such as, plasma glucose, plasma, and tissue MDA levels. MDA, one of the best indicators of oxidative stress [33], is a product of lipid peroxidation and we observed that plasma and tissue MDA levels were significantly increased in D group. However, MDA levels were decreased in the C + Vit B6 and D + Vit B6 groups and this reduction might be attributed to antioxidant effects of vitamin B6. It was suggested that vitamin B6 acts as a powerful chain-breaking antioxidant in biological systems related to its ability to scavenge peroxyl radicals [18, 20, 21]. Furthermore, as observed in the present study, hypocholesterolemic effect of vitamin B6 might also contribute to its antioxidant effect, since lipids are the primary targets of ROS.

Antioxidant enzymes SOD, CAT, and GSH-Px exist in all oxygen-metabolizing cells to prevent cells from damage exerted by free radicals and provide a repair mechanism for oxidized components [34]. SOD dismutases superoxide, the first step generated radical, to hydrogen peroxide and oxygen. Hydrogen peroxide (H_2_O_2_) is neutralized to H_2_O by Gpx or CAT. There is currently no consensus regarding response of antioxidant enzymes in the diabetes mellitus; some studies reported increased [35, 36] and others decreased [37, 38] SOD and/or GSH-Px activity in diabetes. We found increased SOD and GSH-Px activities in D group compared with C group (*P* < 0.05). Increase in the activity of these antioxidant enzymes might be a compensatory defensive response to overproduction of ROS since oxidative damage related to hyperglycemia is predominantly caused by mitochondrial superoxide over production [38]. Compensatory increase of erythrocyte SOD, which acts as a crucial enzyme in scavenging superoxide would therefore be beneficial. Furthermore, erythrocyte SOD and whole blood GSH-Px activities were significantly decreased in D + Vit B6 group compared with D group (Table 2). It is possible that vitamin B6 supplementation eliminated the requirement for the compensatory response of these antioxidant enzymes related to the abovementioned antioxidant properties of vitamin B6. Interestingly, SOD and Gpx activities were increased in the C + Vit B6 group which might suggest a direct stimulating effect of vitamin B6 on the activity of these antioxidant enzymes in normal conditions, since effects of vitamin B6 (or other antioxidant molecules) on antioxidant enzyme activity might depend on the oxidative status of the organism [21–23].

In the present study, serum paraoxonase and arylesterase activities were reduced in the STZ-diabetic rats, which were consistent with previous diabetic human and rat studies [39–43]. Decreased activity of PON-1 can result in disturbances in HDL activity, such as, the ability of circulating HDL particles to protect LDL from oxidation, cholesterol efflux from cells, and inhibition of monocyte-endothelial cell interaction [4, 8, 44]. It was reported that paraoxonase and/or arylesterase activities were reduced in several situations associated with oxidative stress, including diabetes, hyperlipidemia, and coronary artery disease [11–13, 45–47]. Decreased serum paraoxonase activity in diabetic rats might be related to hyperglycemia and/or oxidative stress since the enzyme activity was shown to be reduced related to glycation and glycol oxidation of HDL in the hyperglycemic state. Furthermore, glycosylation or oxidative modification of transcription factors or nucleic material inhibits synthesis of enzyme and it is widely accepted that decreased arylesterase activity, as observed in the present study, reflects reduction in the mass of the enzyme. Serum paraoxonase and arylesterase activities were increased both in the D + Vit B6 and C + Vit B6 groups and might be related to the direct stimulating effect of vit B6 on PON1 and/or its antioxidant properties. Effects of vitamin B6 on PON1 may be one of the antiatherosclerotic activities of this vitamin which deserves further investigation.

In the present study, in line with previous reports [48–50] we observed decrement in serum vitamin B6 levels in STZ-induced diabetic rats compared with those of the control group (*P* < 0.05). There are contradictory reports regarding vitamin B6 levels in the type 1 diabetes mellitus. Leklem and Hollenbeck reported that [24] hyperglycemia induced vitamin B6 deficiency while others [25, 51] suggested that vitamin B6 deficiency induced a decrease in circulating insulin levels which resulted in diabetes. Okada et al. proposed that diabetic rats should have a higher intake of vitamin B6 since diabetic state could lead to a vitamin B6 deficiency and might require vitamin B6 supplementation [27]. Vitamin B6 by acting as a coenzyme for transaminase and glycogen phosphorylase, pyridoxal phosphate (PLP) is involved in gluconeogenesis and glycogenolysis [26, 48, 52]. In this study serum vitamin B6 levels were significantly increased in the C + Vit B6 and D + Vit B6 supplementation groups compared with the C and D groups, respectively (*P* < 0.05).

The results of present study suggest that, besides oxidative stress and other metabolic changes, diabetes is accompanied with reduced levels of vitamin B6 and it seems likely that supplementation of vitamin B6 improves oxidative stress and lipid profile in diabetes. Furthermore, vitamin B6 supplementation enhanced serum paraoxonase and arylesterase activities which might be related to a possible direct effect of this vitamin on the enzyme and/or related to its ability to reduce oxidative stress. Taking the data of this study into consideration vitamin B6 supplementation might be a promising adjunctive agent for improving oxidative stress and metabolic disturbances and for preventing diabetic complications including atherogenesis.

## Figures and Tables

**Table 1:** Body weight, food and water consumption, and metabolic parameters of the control and experimental groups of rats.

Group	C	C + Vit B6	D	D + Vit B6
Food intake (g per 24 h)	18 ± 1	14 ± 1	33 ± 5^a∗^	28 ± 3
Water intake (mL per 24 h)	29 ± 1	36 ± 1	187 ± 9^a∗^	180 ± 5
Final body weight (g)	376 ± 5	363 ± 28	269 ± 27^a∗^	296 ± 14
Glucose (mmol/L)	6.80 ± 0.27	6.98 ± 0.12	22.00 ± 1.37^a∗^	21.43 ± 0.57
TC (mmol/L)	2.55 ± 0.15	1.67 ± 0.11^a∗∗^	3.28 ± 0.14^a∗^	2.44 ± 0.17^b∗^
TG (mmol/L)	0.77 ± 0.03	0.71 ± 0.06	3.07 ± 0.28^a∗∗^	2.98 ± 0.30
HDL-C (mmol/L)	1.29 ± 0.08	1.32 ± 0.01^a∗∗^	1.22 ± 0.05^a∗∗^	1.25 ± 0.01^b∗^

TC: total cholesterol, tG: Triglyceride, and HDL-C: high density lipoprotein-cholesterol.

C: normal control rats, C + Vit B6: control rats with orally administered vitamin B6, D: streptozotocin-induced diabetic rats, and D + Vit B6: diabetic rats with orally administered vitamin B6.

Values are expressed as mean ± S.E.M. for eight rats in each group.

Statistical comparison: ^a^compared with the C group and ^b^compared with the D group.

Statistical significance, ^*^ *P* < 0.05 and ^**^ *P* < 0.01.

**Table 2:** Antioxidative enzyme activities and serum vitamin B6 levels in control and experimental groups of rats.

Group	C	C + Vit B6	D	D + Vit B6
Paraoxonase activity (U/L)	122.11 ± 1.70	153,7 ± 5.78^a∗^	52.30 ± 7.41^a∗∗^	81.17 ± 4.26^b∗^
Arylesterase activity (kU/L)	143.52 ± 8.47	181.01 ± 5.54^a∗^	72.66 ± 6.74^a∗∗^	91.46 ± 2.54^b∗^
GSH-Px (U/mL )	9.9 ± 0.1	17.7 ± 1.7^a∗^	22.5 ± 1.0^a∗^	13.9 ± 1.3^b∗^
SOD (U/mL)	61.3 ± 4.1	88.7 ± 7.5^a∗∗^	112.5 ± 5.7^a∗^	96.9 ± 6.7^b∗^
Vitamin B6 (*µ*g/L)	166.6 ± 23.6	232.45 ± 24.69^a∗^	104.52 ± 1.47^a∗^	145.06 ± 0.65^b∗^

SOD: superoxide dismutase and GSH-Px: glutathione peroxidase.

Statistical comparison: ^a^compared with the C group and ^b^compared with the D group.

Statistical significance, ^*^ *P* < 0.05 and ^**^ *P* < 0.01.

For abbreviations of study groups, see Table 1.
